# The Interplay Between Melatonin and Nitric Oxide: Mechanisms and Implications in Stroke Pathophysiology

**DOI:** 10.3390/antiox14060724

**Published:** 2025-06-13

**Authors:** Santos Blanco, María del Mar Muñoz-Gallardo, Raquel Hernández, María Ángeles Peinado

**Affiliations:** Department of Experimental Biology, University of Jaén, 23007 Jaén, Spain; mmgallar@ujaen.es (M.d.M.M.-G.); rhernand@ujaen.es (R.H.); apeinado@ujaen.es (M.Á.P.)

**Keywords:** melatonin, nitric oxide, stroke, CNS, ischemia, molecular mechanisms

## Abstract

This work reviews the complex interplay between melatonin and nitric oxide (NO) in the central nervous system (CNS), with a detailed focus on its involvement in stroke pathophysiology. Melatonin, a neurohormone with potent antioxidant, anti-inflammatory, and neuroprotective properties, and NO, a gaseous signaling molecule with diverse roles, interact crucially. In the context of ischemic stroke, NO exhibits a dual role: it can be neuroprotective (primarily via endothelial nitric oxide synthase (eNOS)) or neurotoxic (especially through inducible nitric oxide synthase (iNOS) and neuronal nitric oxide synthase (nNOS), contributing to the formation of damaging peroxynitrite (ONOO^−^)). Melatonin has consistently demonstrated neuroprotective effects in animal models of stroke. Its key mechanisms related to NO include (1) differential modulation of nitric oxide synthase isoforms, suppressing detrimental iNOS expression/activity while often preserving or enhancing beneficial eNOS; (2) direct scavenging of NO and, critically, highly reactive peroxynitrite, thereby attenuating nitrosative stress; (3) reduction in neuroinflammation, partly by promoting M2 (anti-inflammatory) microglia polarization; and (4) mitochondrial protection and decreased apoptosis. These multifaceted actions of melatonin contribute to reduced infarct volume and improved functional outcomes, underscoring its considerable therapeutic potential for ischemic stroke through the favorable modulation of the melatonin–NO axis.

## 1. Introduction

### 1.1. Overview of Melatonin

Melatonin (N-acetyl-5-methoxytryptamine) is a phylogenetically ancient and remarkably conserved molecule found across diverse biological kingdoms, including animals [1]. In vertebrates, it is most recognized as a neurohormone primarily synthesized and rhythmically secreted by the pineal gland, predominantly during the nocturnal phase [1]. This rhythmic secretion, driven by the central circadian clock located in the suprachiasmatic nucleus (SCN) and regulated by environmental light–dark cycles via sympathetic innervation of the pineal gland, serves as a crucial endocrine signal for synchronizing daily and seasonal physiological rhythms [2]. The synthesis pathway in animals originates from the essential amino acid tryptophan, proceeding through serotonin via the enzymes tryptophan hydroxylase (TPH), aromatic L-amino acid decarboxylase (AADC), arylalkylamine N-acetyltransferase (AANAT), and acetylserotonin O-methyltransferase (ASMT, also known as HIOMT) [3]. Beyond its chronobiotic functions, extensive research, particularly within the last two decades, has unveiled a wide array of pleiotropic effects exerted by melatonin. These include potent antioxidant actions, significant anti-inflammatory and immunomodulatory properties, and well-documented neuroprotective capabilities, making it a molecule of considerable interest in various physiological and pathological contexts [4]. Recent studies have also highlighted its role in regulating energy metabolism and mitochondrial homeostasis, further expanding its therapeutic relevance [5].

One of the most advantageous pharmacokinetic properties of melatonin is its amphiphilic nature, which allows it to easily cross all biological barriers, including the blood–brain barrier (BBB), and distribute widely in all cellular compartments (membrane, cytosol, mitochondria, and nucleus) [6].

The neuroprotective actions of melatonin are diverse and can be grouped into several main categories:

- Antioxidant mechanisms: Melatonin is a potent antioxidant. It acts as a direct scavenger of a wide range of reactive oxygen species (ROS), such as the hydroxyl radical (^•^OH) and the superoxide anion O_2_^•−^, and reactive nitrogen species (RNS), including peroxynitrite (ONOO^−^) [7]. Importantly, its main metabolites, such as N1-acetyl-N2-formyl-5-methoxykynuramine (AFMK) and N1-acetyl-5-methoxykynuramine (AMK), also possess notable antioxidant activity, which amplifies and prolongs its protective effect [6]. In addition to direct scavenging, melatonin exerts indirect antioxidant effects by stimulating the activity of key endogenous antioxidant enzymes, such as superoxide dismutase (SOD), glutathione peroxidase (GPx), glutathione reductase (GR), and catalase [7]. It also enhances the nuclear factor erythroid 2-related factor 2 (Nrf2) pathway, a master transcription factor that regulates the expression of multiple antioxidant defense and detoxification genes [8].
- Anti-inflammatory actions: Melatonin exerts potent anti-inflammatory effects. It reduces the production and release of pro-inflammatory cytokines such as tumor necrosis factor-alpha (TNF-α), interleukin-1 beta, and interleukin-6 (IL-6) [6]. It modulates the activation of microglia, the brain’s resident immune cells, potentially promoting a shift from a pro-inflammatory (M1) phenotype to an anti-inflammatory and pro-repair (M2) phenotype [8]. It also inhibits key pro-inflammatory signaling pathways, such as that of nuclear factor kappa B (NF-κB), and reduces the expression of cell adhesion molecules involved in leukocyte infiltration into damaged brain tissue [9].
- Anti-apoptotic effects: Melatonin interferes with the apoptotic cascade at multiple levels. One of its crucial sites of action is the mitochondria, where it prevents the opening of the mitochondrial permeability transition pore (mPTP), an event that leads to the dissipation of the mitochondrial membrane potential and the release of pro-apoptotic factors [7]. Consequently, melatonin reduces the release of cytochrome c from the mitochondria to the cytosol and decreases the activation of caspases, the executioner enzymes of apoptosis [7]. Furthermore, it modulates the expression of Bcl-2 family proteins, increasing the levels of anti-apoptotic proteins (e.g., Bcl-2) and decreasing those of pro-apoptotic proteins (e.g., Bax) [7]. It also activates pro-survival signaling pathways, such as the PI3K/Akt pathway [10].

Melatonin exerts many of its effects through interaction with two main types of G protein-coupled membrane receptors, designated MT1 and MT2 [10]. However, a significant part of its actions, especially its direct antioxidant effects, is independent of these receptors and is due to its intrinsic chemical properties [10]. This duality in its mechanism of action (receptor-dependent and -independent) could confer particular robustness as a therapeutic agent, allowing it to act even when receptor expression might be altered by pathology.

### 1.2. Overview of Nitric Oxide (NO)

Nitric oxide (NO) is another fundamental signaling molecule in animals, unique in its nature as a gaseous free radical [11]. It is enzymatically generated from the amino acid L-arginine by a family of enzymes known as nitric oxide synthases (NOSs) [12]. NO participates in an impressive spectrum of physiological processes across multiple organ systems. Its roles include the regulation of vascular tone and blood pressure, neurotransmission within the central and peripheral nervous systems, modulation of immune responses and host defense against pathogens, and regulation of energy metabolism [11]. The diverse functions of NO stem from its ability to readily diffuse across cell membranes and interact with various molecular targets, most notably activating soluble guanylyl cyclase (sGC) to produce cyclic GMP (cGMP), but also interacting with heme proteins and mediating post-translational modifications like S-nitrosylation [11].

As a small, lipophilic gaseous molecule, NO is not stored in synaptic vesicles or released by exocytosis, but is synthesized “on demand” and diffuses freely across cell membranes, acting as a paracrine or “volume” messenger on neighboring cells, both neuronal and non-neuronal, within a limited radius determined by its short half-life and reactivity [13]. Control of NO synthesis is, therefore, the key point for regulating its activity.

### 1.3. Significance of Melatonin–NO Interactions in the Central Nervous System

The central nervous system (CNS) represents a critical arena for the interaction between melatonin and nitric oxide pathways. The brain’s high metabolic rate, substantial oxygen consumption, and lipid-rich composition render it particularly vulnerable to damage from ROS and RNS, including NO and its derivatives [14]. Furthermore, the CNS possesses relatively limited endogenous enzymatic defenses against oxidative stress compared to other tissues [14]. Within this environment, the interplay between melatonin and NO significantly influences neuronal function, synaptic plasticity, cerebrovascular regulation, neuroinflammation, and ultimately, cell survival or death under both physiological and pathological conditions [6]. Melatonin’s relevance to CNS processes is significantly enhanced by its amphiphilic nature, which allows it to readily cross the BBB and distribute widely within neural tissues, reaching both neuronal and glial cells, as well as subcellular compartments like mitochondria [6]. Concentrations of melatonin in the cerebrospinal fluid (CSF) can be substantially higher than in the plasma, further supporting its direct actions within the brain [6]. The co-regulation between melatonin and NO is crucial for maintaining optimal neuronal function, especially in protecting against oxidative stress and excitotoxicity in brain aging [15].

### 1.4. Rationale and Scope

Given the crucial roles of both melatonin and nitric oxide in CNS function and the brain’s susceptibility to imbalances in their signaling, understanding their interaction is paramount, especially in the context of neurological diseases. Stroke, a major cause of mortality and disability worldwide, involves a complex pathophysiology where both oxidative/nitrosative stress and inflammation, processes heavily influenced by both melatonin and NO, play central roles [14]. This review aims to provide an insight into the relationship between melatonin and nitric oxide specifically within the central nervous system, with a detailed focus on the context of stroke.

## 2. Melatonin and Nitric Oxide Signaling in the Animal CNS

### 2.1. Melatonin in the CNS

#### 2.1.1. Synthesis and Distribution

The primary source of circulating melatonin in mammals is the pineal gland, where its synthesis is tightly regulated by the light–dark cycle [1]. Light information perceived by the retina is relayed via the retinohypothalamic tract to the SCN, the master circadian pacemaker. The SCN, through a multi-synaptic pathway involving the paraventricular nucleus and the superior cervical ganglion, controls the nocturnal release of norepinephrine onto pinealocytes [1]. This neurotransmitter activates adrenergic receptors, primarily β1 and α1, leading to increased intracellular cAMP levels and subsequent activation of protein kinase A (PKA) [16]. PKA plays a crucial role in regulating AANAT, the rate-limiting enzyme in melatonin synthesis in many species [17,18], primarily through post-translational mechanisms (preventing proteasomal degradation via phosphorylation and binding to 14-3-3 proteins) in ungulates and primates, and additionally through transcriptional activation (via CREB phosphorylation) in rodents [16]. ASMT then catalyzes the final step, converting N-acetylserotonin (NAS) to melatonin [19].

While the pineal gland dictates circulating melatonin rhythms, evidence suggests melatonin synthesis also occurs in various extrapineal sites, including within the CNS itself [3]. The retina, particularly in photoreceptor cells, exhibits rhythmic melatonin production under circadian and photic control, although this retinal melatonin primarily acts locally and does not significantly contribute to circulating levels [20]. Studies have also detected the necessary enzymes (AANAT and ASMT/HIOMT) and melatonin itself in various brain regions, including the hypothalamus and potentially astrocytes, suggesting local, non-circadian production might occur [19]. Melatonin synthesis in the gut, as well as its ability to influence the gut–brain axis, is also an emerging area of research with implications for CNS health [21]. Critically, melatonin synthesis has been proposed to occur within mitochondria in virtually all cells, including those in the CNS [3]. This subcellular localization is significant given mitochondria are major sites of ROS production and melatonin’s potent antioxidant function [3]. Mitochondrial melatonin has been shown to be fundamental in protecting against oxidative damage and mitochondrial dysfunction in neurodegenerative diseases [22]. Melatonin readily enters the CNS from the circulation, achieving notable concentrations in the CSF [6]. Within the brain, melatonin undergoes metabolism, partly via cytochrome P450 enzymes (isoforms like CYP1A1, CYP1A2, CYP1B1, and CYP2C19 are present in the brain) leading to 6-hydroxymelatonin, but also through oxidative pyrrole-ring cleavage, generating metabolites like AFMK and AMK, which are themselves biologically active, particularly as antioxidants [19].

#### 2.1.2. Receptors

Melatonin exerts many of its physiological effects through interactions with specific high-affinity membrane receptors, primarily MT1 (Mel1a) and MT2 (Mel1b) [1]. Both are members of the G protein-coupled receptor (GPCR) superfamily and are widely distributed throughout the CNS, albeit with distinct patterns [19]. MT1 receptors are generally more abundant and found in areas like the SCN, hippocampus, cerebellum, *substantia nigra pars reticulata*, thalamus, and cortical regions [19]. MT2 receptors are also present in the SCN, retina, hippocampus (particularly CA3), thalamic reticular nucleus, and other areas [19]. Their expression in both neurons and glial cells underscores melatonin’s broad influence within the brain [19]. Activation of MT1 and MT2 receptors typically couples to inhibitory G proteins (Gi/o), leading to the inhibition of adenylyl cyclase and a decrease in intracellular cAMP levels [2]. However, they can also couple to other G proteins (e.g., Gq/11) to activate phospholipase C (PLC), modulating intracellular calcium levels and protein kinase C (PKC) activity, or influence ion channel function and pathways like the MEK/ERK cascade [23]. These signaling events ultimately regulate gene expression and cellular function, mediating melatonin’s effects on circadian rhythms, neuronal activity, and neuroprotection [24]. It is important to note that receptor affinities and the relative importance of MT1 versus MT2 can vary between species, a factor relevant for translating findings from animal models [25]. Recent studies have identified complex interactions between MT1 and MT2 receptors and other signaling systems in the brain, such as the endocannabinoid system, which could have therapeutic implications [26]. Besides membrane receptors, melatonin may interact with the orphan nuclear receptors RZR/ROR and the cytosolic enzyme quinone reductase 2 (QR2, sometimes referred to as MT3), potentially mediating some of its antioxidant and other effects, although the physiological significance of these interactions is still under investigation.

#### 2.1.3. Key CNS Functions (Beyond Circadian)

While regulation of circadian and seasonal rhythms remains a primary function, melatonin’s roles within the CNS extend far beyond chronobiology. Its neuroprotective actions are extensively documented across various models of neurological injury and disease [4]. These protective effects are mediated through both receptor-dependent signaling pathways and receptor-independent mechanisms, primarily its potent antioxidant and free radical scavenging activity [3]. Melatonin has been shown to be effective in modulating synaptic plasticity and memory formation, underscoring its potential in the treatment of cognitive disorders [27]. Melatonin also modulates neuroinflammation, influences neurotransmitter systems (e.g., GABAergic, glutamatergic), affects neurogenesis and neuroplasticity, and may play roles in mood regulation and energy balance within the CNS [1].

### 2.2. Nitric Oxide in the CNS

#### 2.2.1. Synthesis (NOS Isoforms)

Nitric oxide production in the CNS, as elsewhere in the body, is catalyzed by three distinct isoforms of nitric oxide synthase (NOS) [28]. These isoforms, encoded by separate genes, are neuronal NOS (nNOS or NOS1), inducible NOS (iNOS or NOS2), and endothelial NOS (eNOS or NOS3) [29]. Both nNOS and eNOS are termed constitutive (cNOS) as they are typically present under basal conditions and their activity is acutely regulated by intracellular calcium (Ca^2+^) levels, mediated via the binding of Ca^2+^-calmodulin (CaM). In contrast, iNOS expression is normally very low but is strongly upregulated by pro-inflammatory stimuli like cytokines (e.g., TNF-α, IL-1β, IFN-γ) and bacterial lipopolysaccharide (LPS) [29]. Once expressed, iNOS produces much larger quantities of NO for sustained periods and its activity is largely independent of fluctuations in intracellular Ca^2+^ due to its very tight binding to CaM [30]. All three isoforms catalyze the same reaction: the five-electron oxidation of a guanidino nitrogen of L-arginine to produce L-citrulline and NO [31]. This complex reaction requires molecular oxygen (O_2_) and several cofactors, including NADPH, FAD, FMN, and tetrahydrobiopterin (BH4) [28]. The enzymes function as homodimers, with each monomer containing an N-terminal oxygenase domain (binding heme, BH4, and L-arginine) and a C-terminal reductase domain (binding NADPH, FAD, and FMN), linked by a CaM-binding region [31]. Within the CNS, nNOS is predominantly found in specific neuronal populations throughout the brain and spinal cord [32]. eNOS is primarily localized to the vascular endothelium lining cerebral blood vessels, but may also be present in some neuronal populations [28]. iNOS is typically expressed in microglia, astrocytes, and infiltrating macrophages following injury or inflammatory challenge [28]. Additionally, evidence suggests the existence of functional NOS activity within mitochondria, which appears related to both nNOS and iNOS isoforms and may play a role in regulating mitochondrial respiration and ROS production [33]. The interaction of mitochondrial NO with key electron transport chain proteins is an important mechanism in the regulation of cellular energy metabolism [34].

#### 2.2.2. Signaling

The primary and best-characterized signaling mechanism for NO involves the activation of soluble guanylyl cyclase (sGC) in target cells [11]. NO binds to the heme moiety of sGC, causing a conformational change that dramatically increases the enzyme’s activity, leading to the conversion of GTP to the second messenger cyclic GMP (cGMP) [12]. Elevated cGMP levels then activate downstream effectors, most notably cGMP-dependent protein kinase (PKG), which phosphorylates various target proteins to elicit specific cellular responses, such as smooth muscle relaxation [35]. NO can also exert effects through cGMP-independent mechanisms, including direct modification of proteins via S-nitrosylation (the covalent attachment of an NO group to a cysteine thiol) [36]. S-nitrosylation can alter protein function, localization, and stability, affecting a wide range of cellular processes, including ion channel activity, receptor function, and enzyme regulation. Other potential interactions include direct reactions with metal centers in proteins or lipids.

#### 2.2.3. Physiological Roles

In the CNS, NO generated by different NOS isoforms plays distinct and crucial roles. nNOS-derived NO acts as a neurotransmitter or neuromodulator, involved in processes like synaptic plasticity (e.g., long-term potentiation in the hippocampus, relevant for learning and memory), regulation of cerebral blood flow through neurovascular coupling, and central control of autonomic functions like blood pressure [32]. eNOS-derived NO is the principal mediator of endothelium-dependent vasodilation in the cerebral circulation, crucial for maintaining adequate blood flow and responding to changes in metabolic demand [11]. It also possesses anti-atherogenic properties by inhibiting platelet aggregation and leukocyte adhesion to the endothelium [31]. iNOS-derived NO, produced in large amounts during neuroinflammation (e.g., infection, injury), primarily serves a host defense function by exerting cytotoxic/cytostatic effects against pathogens and tumor cells [32]. However, this high-output NO production can also contribute to neuronal damage and exacerbate pathology in inflammatory and neurodegenerative conditions [32]. NO also participates in the regulation of cellular energy metabolism, influencing mitochondrial respiration and the utilization of glucose and fatty acids, with effects dependent on NO concentration and cellular context [12]. Dysfunction in NO signaling, particularly eNOS dysregulation, has been associated with the development and progression of neurovascular diseases [37].

As indicated, NO is key in synaptic plasticity and therefore in learning and memory processes, which are severely affected by ischemic stroke. More specifically, NO is widely recognized as a crucial mediator in various forms of synaptic plasticity, especially in NMDA receptor-dependent long-term potentiation (LTP) in regions such as the hippocampus and neocortex [38]. In this context, NO often functions as a retrograde messenger: following postsynaptic activation of NMDA receptors and the consequent Ca^2+^ influx, nNOS (coupled to NMDA receptors via PSD-95) is activated, producing NO that diffuses to the presynaptic terminal [13]. There, NO, mainly through the sGC-cGMP-PKG pathway, can enhance neurotransmitter release, contributing to LTP expression [39]. In addition to its role in LTP, NO has also been implicated in certain forms of long-term depression (LTD) and in homeostatic plasticity. S-nitrosylation of synaptic proteins, such as subunits of NMDA or AMPA receptors and presynaptic proteins, also contributes to the fine-tuning of synaptic transmission and plasticity [38]. Given its fundamental involvement in the cellular mechanisms of plasticity, it is not surprising that NO plays an important role in learning and memory processes. Pharmacological studies with NOS inhibitors or NO donors, as well as studies with genetically modified mice for NOS isoforms, have consistently shown that alteration of NO signaling can affect performance in various spatial learning, avoidance, and recognition memory tasks [40].

### 2.3. Molecular Crosstalk Between Melatonin and NO/NOS in the CNS

The interaction between melatonin and the nitric oxide system in the CNS is multifaceted, involving regulation of NOS enzymes, modulation of NO signaling, and direct chemical interactions. This crosstalk results in complex physiological outcomes that are highly dependent on the specific context, including the physiological state (e.g., health vs. disease), the cell type involved, and the relative concentrations of the signaling molecules.

#### 2.3.1. Regulation of NOS Expression and Activity

Melatonin exerts differential effects on the expression and activity of the three main NOS isoforms.

iNOS: A consistent finding across numerous studies is that melatonin inhibits the induction and/or activity of iNOS, particularly in response to pro-inflammatory stimuli (like LPS or cytokines) or pathological conditions such as ischemia/reperfusion or hypoxia [41]. Several molecular mechanisms underpin this inhibition. Melatonin can suppress the activation of the transcription factor NF-κB, a key regulator of the iNOS gene promoter [42]. This involves, at least in part, melatonin’s ability to inhibit the histone acetyltransferase (HAT) activity of p300, which reduces the acetylation of the p52 NF-κB subunit, thereby decreasing its binding to the iNOS promoter [42]. Additionally, melatonin can inhibit the p38 MAPK pathway, which is also involved in cytokine-induced iNOS expression in glial cells [43]. This suppression of iNOS-mediated NO production is a major component of melatonin’s anti-inflammatory and neuroprotective effects [41]. Melatonin has been observed to reduce iNOS expression in models of chronic inflammation, suggesting therapeutic potential in neurodegenerative diseases characterized by neuroinflammation [44].

nNOS: The regulation of nNOS by melatonin appears more complex and context-dependent. Some studies report that melatonin can inhibit nNOS activity, potentially through direct interaction with the enzyme or by interfering with its activation by CaM [45]. In inflammatory pain models, melatonin reversed capsaicin-induced upregulation of nNOS in sensory neurons [46]. In the dorsal raphe nucleus (DRN) of rats, melatonin treatment suppressed nNOS expression, contributing to its effects on wakefulness signaling [47]. However, in the context of cerebral ischemia, the effects are varied. Some studies found that melatonin pre-treatment significantly lessened the ischemia/reperfusion-induced increase in nNOS immunoreactivity [48] or prevented the increase altogether [49]. Conversely, another study using global cerebral ischemia found higher nNOS expression in post-ischemia melatonin-treated rats compared to pre-ischemia treated rats, although lower than in untreated ischemic animals [50]. Yet another study reported that melatonin does not influence the nNOS increase caused by oxygen treatment following ischemia [51]. Furthermore, one in vitro study showed that nanomolar concentrations of melatonin could transiently increase nNOS mRNA and protein expression in HaCat cells [52]. This variability underscores that melatonin’s effect on nNOS likely depends on the specific physiological or pathological state, the cell type, the timing and concentration of melatonin, and the specific regulatory mechanisms dominant in that context.

eNOS: Melatonin’s interaction with eNOS is also nuanced. In pathological conditions like cerebral ischemia or in specific brain regions of hypertensive rats, melatonin treatment has been shown to prevent the injury-induced decrease in eNOS expression or even increase eNOS expression and/or activity [49]. This preservation or upregulation of eNOS could contribute to neuroprotection by maintaining beneficial vasodilation and endothelial function. Potential mechanisms include activation of pro-survival pathways like Akt [51] or stabilization of the eNOS enzyme complex [53]. However, other studies suggest inhibitory interactions. For instance, melatonin has been proposed to inhibit eNOS activity via modification of the Ca^2+^-CaM complex [54], similar to its potential effect on nNOS. Furthermore, in porcine coronary arteries, melatonin inhibits NO-mediated relaxation by activating PKG1, which phosphorylates and increases the activity of phosphodiesterase 5 (PDE5), leading to enhanced cGMP degradation [35]. While this specific mechanism was identified in coronary arteries, it highlights a potential pathway for melatonin to counteract eNOS-derived NO signaling in certain vascular beds or conditions. New research suggests that melatonin can restore eNOS function under oxidative stress conditions by improving the bioavailability of BH4, an essential cofactor for eNOS activity [55].

In addition, melatonin appears to play a crucial role in regulating NO production within mitochondria. It has been shown to protect mitochondria from excessive NO levels that can inhibit respiration and increase oxidative stress [33]. This regulation is vital for maintaining mitochondrial homeostasis and bioenergetic function [33].

#### 2.3.2. Impact on NO Bioavailability and Downstream Signaling

Through its differential regulation of NOS isoforms, melatonin significantly influences the overall bioavailability of NO, particularly under pathological conditions where it often curtails excessive production, primarily from iNOS [56]. This modulation impacts downstream NO signaling pathways. As mentioned, melatonin can inhibit NO-induced cGMP accumulation in specific contexts, such as via PDE5 activation in coronary smooth muscle [35]. This contrasts with the typical vasodilatory effect mediated by the NO-sGC-cGMP pathway [12]. A specific example of melatonin modulating an NO-dependent pathway for physiological regulation occurs in the rat DRN. Here, melatonin (acting during the night when its levels are high in rats) suppresses nNOS expression. This leads to increased expression of WNK4 kinase, which subsequently increases the phosphorylation of OSR1 and the expression of the NKCC1 chloride co-transporter. The resulting potential increase in intracellular chloride concentration in wake-promoting serotonergic neurons may attenuate GABAergic inhibition, thereby promoting wakefulness [47]. This detailed pathway illustrates how melatonin can intricately regulate CNS function by intervening in NO-dependent signaling cascades, demonstrating a sophisticated modulatory role rather than simple inhibition.

#### 2.3.3. Direct Scavenging of NO and Reactive Nitrogen Species

Beyond regulating NOS enzymes, melatonin possesses remarkable receptor-independent antioxidant properties, including the direct scavenging of NO radicals [57]. Perhaps even more critically, melatonin is an exceptionally potent scavenger of ONOO^−^, a highly reactive and cytotoxic RNS [57]. Peroxynitrite is formed via a diffusion-limited reaction between NO and O_2_^•−^, both of which can be excessively produced under conditions of oxidative stress and inflammation, such as during stroke [36]. By effectively neutralizing ONOO^−^, melatonin prevents downstream deleterious events, including lipid peroxidation, protein nitration (formation of nitrotyrosine, a marker of ONOO^−^ damage), DNA damage, and mitochondrial dysfunction [58]. Melatonin also detoxifies other harmful species derived from NO/ONOO^−^ reactions, such as nitrogen dioxide radicals (^•^NO_2_), hydroxyl radicals (^•^OH), and carbonate radicals (CO_3_^•−^) [36]. An important feature of melatonin’s antioxidant action is the formation of metabolites (AFMK and AMK) during these scavenging reactions, which are themselves potent antioxidants, thus creating a cascade effect that amplifies its protective capacity [3]. This direct scavenging activity complements its ability to regulate NOS expression and activity, providing a robust, multi-pronged defense against nitrosative stress. Under conditions of high RNS production, such as stroke, where enzymatic defenses may be overwhelmed, this direct detoxification capacity is likely essential for melatonin’s neuroprotective efficacy.

## 3. The Melatonin–NO Axis in Stroke Pathophysiology and Neuroprotection

### 3.1. Overview of Stroke Pathophysiology

Ischemic stroke results from a sudden interruption of blood flow to a region of the brain, typically due to thrombosis or embolism occluding a cerebral artery [9]. This cessation of flow rapidly leads to oxygen and glucose deprivation (hypoxia/anoxia), causing energy failure (ATP depletion) within the affected neural tissue [9]. If blood flow is not restored promptly, irreversible neuronal damage occurs in the ischemic core. The surrounding area, the penumbra, experiences less severe blood flow reduction and remains potentially salvageable for a limited time [59]. Paradoxically, the restoration of blood flow (reperfusion), while necessary for tissue survival, triggers a secondary cascade of damaging events known as ischemia/reperfusion (I/R) injury [8]. This I/R injury significantly contributes to the final extent of brain damage. The complex pathophysiology involves multiple interconnected processes:

- Excitotoxicity: Excessive release of excitatory neurotransmitters, primarily glutamate, overstimulates receptors (especially NMDA receptors), leading to massive Ca^2+^ influx [60].
- Ionic imbalance and calcium overload: Energy failure disrupts ion pumps, leading to loss of ionic gradients and toxic intracellular Ca^2+^ accumulation [61].
- Oxidative and nitrosative stress: I/R dramatically increases the production of ROS and RNS from various sources (mitochondria, NADPH oxidases, uncoupled NOS, xanthine oxidase), overwhelming antioxidant defenses and causing widespread molecular damage [8].
- Neuroinflammation: Rapid activation of resident microglia and astrocytes, followed by infiltration of peripheral immune cells (neutrophils, macrophages), releases pro-inflammatory cytokines (TNF-α, IL-1β, IL-6), chemokines, and matrix metalloproteinases (MMPs), further propagating tissue injury [8].
- Mitochondrial dysfunction: Mitochondria are central players, suffering damage from Ca^2+^ overload and oxidative/nitrosative stress, leading to impaired ATP production, increased ROS generation, opening of mPTP, and release of pro-apoptotic factors [61].
- Cell death: Neurons and glial cells die via various mechanisms, including necrosis (primarily in the core), apoptosis (prominent in the penumbra), and potentially other regulated cell death pathways like pyroptosis and necroptosis, particularly exacerbated in conditions like obesity [8]. New research has identified ferroptosis as an additional mechanism of cell death in ischemic stroke, and melatonin may play a role in its modulation [62].
- Blood–brain barrier (BBB) breakdown: Increased permeability of the BBB allows influx of fluid and peripheral immune cells, contributing to cerebral edema and inflammation [8].

### 3.2. The Complex Role of NO and NOS Isoforms in Stroke

Nitric oxide exhibits a dichotomous role in the setting of ischemic stroke, with actions that can be either neuroprotective or neurotoxic depending on the NOS isoform source, the concentration and location of NO production, the timing relative to the ischemic insult, and the surrounding biochemical environment (e.g., redox state) [32].

#### 3.2.1. Detrimental Roles:

- iNOS: Upregulation of iNOS in microglia, macrophages, and astrocytes, driven by the inflammatory response to ischemia, leads to sustained high-level NO production [48]. This excessive NO contributes significantly to cytotoxicity, neuroinflammation, and oxidative/nitrosative stress [32]. High NO concentrations can directly inhibit mitochondrial respiration and react with superoxide to form the highly damaging ONOO^−^ [36]. Studies often show increased iNOS expression correlating with larger infarct volumes and worse outcomes [49].
- nNOS: While nNOS has physiological roles, its overactivation during the excitotoxic phase of stroke, triggered by Ca^2+^ influx via NMDA receptors, can also contribute to neuronal damage [32]. nNOS-derived NO can contribute to ONOO^−^ formation and downstream neurotoxicity. Increased nNOS expression or activity is often observed in the peri-infarct regions following ischemia [35].
- Peroxynitrite formation: The reaction between NO (from any source, but particularly high levels from iNOS/nNOS) and superoxide (abundantly produced during I/R) generates ONOO^−^, a potent oxidant and nitrating agent that damages lipids, proteins, and DNA, contributing significantly to cell death and BBB breakdown [63].

#### 3.2.2. Protective Roles:

- eNOS: NO produced by eNOS in the cerebrovascular endothelium plays a generally protective role by promoting vasodilation, which helps maintain collateral blood flow to the ischemic penumbra, potentially limiting infarct expansion [32]. eNOS-derived NO also has anti-platelet and anti-leukocyte adhesion properties, further supporting microvascular perfusion [31]. However, during I/R, eNOS function can become impaired or “uncoupled” due to cofactor (BH4) oxidation, leading to reduced NO production and increased superoxide generation, thus losing its protective capacity [64]. Some studies report decreased eNOS levels or activity after ischemia [49].

Therefore, therapeutic strategies targeting NO in stroke must consider this complexity, aiming to inhibit detrimental NO production (mainly iNOS, possibly excessive nNOS) while preserving or enhancing beneficial eNOS function.

### 3.3. Melatonin as a Neuroprotective Agent in Animal Stroke Models

A substantial body of preclinical evidence consistently demonstrates the neuroprotective efficacy of melatonin administration in various animal models of ischemic stroke. These models typically involve inducing focal ischemia, often through middle cerebral artery occlusion (MCAO) for varying durations (e.g., 30–90 min) followed by reperfusion, or global cerebral ischemia, in species such as rats and mice [9]. In vitro models using oxygen-glucose deprivation (OGD) followed by reoxygenation in neuronal or glial cell cultures also support these findings [25].

Administration of melatonin, typically via intraperitoneal injection at doses ranging from approximately 4 mg/kg to 20 mg/kg, has shown significant benefits whether given before the ischemic insult (pre-treatment) or, more clinically relevantly, after the onset of ischemia or reperfusion (post-treatment) [9]. Some studies indicate a dose-dependent effect, with higher doses (e.g., 10–20 mg/kg) often providing greater protection than lower doses (e.g., 5 mg/kg) [65].

The observed neuroprotective outcomes are multifaceted and address several key aspects of stroke pathology:

- Reduced infarct volume: Melatonin treatment consistently leads to a significant reduction in the size of the brain infarct in both cortical and subcortical regions [8].
- Decreased cerebral edema: Melatonin helps alleviate brain swelling, a dangerous complication of stroke [8].
- Improved neurological function: Animals treated with melatonin exhibit better performance on various sensorimotor and neurological deficit scales, indicating functional recovery [8].
- Attenuated BBB breakdown: Melatonin often reduces the increased permeability of the BBB following I/R, limiting edema and immune cell infiltration [8]. However, one study using a specific pre-treatment regimen did not observe an effect on Evans blue extravasation at 3 h post-reperfusion, suggesting timing and specific endpoints might influence this outcome [65].
- Increased cell survival: melatonin promotes the survival of neurons and potentially other brain cells within the ischemic territory [66].
- Improved cerebral blood flow (CBF): Some evidence suggests melatonin can improve post-ischemic CBF in the core and penumbra regions [51]. Furthermore, it has been proposed that melatonin can improve cerebral microcirculation and perfusion of tissue at risk in the ischemic penumbra [67].

This robust preclinical evidence strongly supports melatonin’s potential as a therapeutic agent for ischemic stroke [6]. Studies involving pinealectomy (removal of the primary source of endogenous melatonin) further support its role, showing that pinealectomized animals exhibit larger infarcts after MCAO, suggesting endogenous melatonin provides a degree of baseline protection [9].

### 3.4. Cellular and Molecular Mechanisms of Melatonin–NO Interaction in Stroke Neuroprotection

The neuroprotective effects of melatonin in stroke arise from its ability to target multiple pathological pathways simultaneously, with the modulation of the NO system being a central component.

- Modulation of NOS isoforms: As discussed previously, melatonin exerts a beneficial regulatory effect on NOS isoforms in the context of ischemia. It consistently suppresses the detrimental upregulation of iNOS induced by I/R injury [48]. This action directly reduces the production of large amounts of cytotoxic NO associated with neuroinflammation. Simultaneously, melatonin often prevents the ischemia-induced downregulation or dysfunction of the protective eNOS isoform, helping to maintain beneficial endothelial function and potentially improving microvascular perfusion in the penumbra [49]. The effect on nNOS appears less critical or more variable in stroke models treated with melatonin [48]. By differentially regulating these isoforms, melatonin shifts the balance of NO production away from excessive, damaging levels towards a profile more conducive to tissue survival.
- Attenuation of oxidative and nitrosative stress: This is a cornerstone of melatonin’s neuroprotection. By reducing excessive NO production (mainly via iNOS inhibition) [65] and potently scavenging the highly toxic ONOO^−^ formed from the reaction of NO with superoxide [60], melatonin significantly mitigates nitrosative stress. This is evidenced by reduced levels of nitrotyrosine, a footprint of ONOO^−^ damage, in melatonin-treated animals post-stroke [68]. This action prevents downstream damage, including lipid peroxidation [58] and protein oxidation/nitration [36]. These effects are synergistic with melatonin’s well-established broader antioxidant actions, which include direct scavenging of various ROS (e.g., ^•^OH, H_2_O_2_) and indirect enhancement of endogenous antioxidant defenses by stimulating enzymes like SOD, catalase (CAT), and GPx, and maintaining glutathione (GSH) levels [19].
- Anti-inflammatory effects: Neuroinflammation is a major driver of secondary injury after stroke, and melatonin effectively dampens this response [8]. A key mechanism involves modulating the activation state of microglia and infiltrating macrophages. Melatonin promotes a shift from the detrimental, pro-inflammatory M1 phenotype (characterized by production of TNF-α, IL-1β, IL-6, and often high iNOS expression) towards the beneficial, anti-inflammatory and tissue-reparative M2 phenotype [69]. This phenotypic switch leads to reduced levels of pro-inflammatory cytokines and MMPs, and potentially increased levels of anti-inflammatory mediators like IL-10 [70]. The signaling pathways mediating this effect include STAT3, which is enhanced by melatonin in microglia exposed to ischemic conditions, and the JAK2-STAT3 pathway [71]. Blockade of STAT3 diminishes melatonin’s ability to induce this phenotype shift [72]. Furthermore, melatonin’s inhibition of the NF-κB pathway contributes to reduced expression of multiple inflammatory genes, including iNOS [9]. Melatonin also reduces the infiltration of peripheral immune cells into the ischemic brain tissue [9].
- Anti-apoptotic mechanisms: Melatonin significantly reduces neuronal apoptosis, a major form of cell death in the ischemic penumbra [73]. Central to this effect is the protection of mitochondria [61]. Melatonin preserves mitochondrial membrane potential (ΔΨm), inhibits the opening of the mPTP (a critical event leading to cell death), reduces the release of pro-apoptotic factors like cytochrome c from mitochondria into the cytosol, maintains the function of electron transport chain (ETC) complexes (particularly complex I and IV), and decreases mitochondrial ROS/RNS generation [61]. It achieves this partly through its direct antioxidant actions within the mitochondria and by modulating signaling pathways. Melatonin also regulates the balance of Bcl-2 family proteins, increasing the expression of anti-apoptotic members like Bcl-2 and Bcl-xL while decreasing pro-apoptotic members like Bax [7]. Consequently, melatonin inhibits the activation of downstream executioner caspases, such as caspase-3 [8]. Additionally, melatonin has been shown to protect against endoplasmic reticulum (ER) stress, another pathway contributing to cell death after ischemia [61].
- Regulation of specific signaling pathways: The neuroprotective effects of melatonin in stroke, including its interplay with NO, are mediated via the modulation of several key intracellular signaling pathways that act as convergent hubs:
  ○ Akt/PI3K pathway: Frequently activated by melatonin, this pathway promotes cell survival by phosphorylating and inactivating pro-apoptotic proteins (like Bad and caspase-9) and activating transcription factors involved in survival and antioxidant responses. Its activation is linked to reduced apoptosis and improved mitochondrial function in stroke models treated with melatonin [8]. Melatonin may activate Akt via MT2 receptors [8] or by suppressing PTEN activity [69]. The Akt-SIRT3-SOD2 axis is specifically implicated in melatonin’s protection against mitochondrial impairment in diabetic stroke models [66].
  ○ STAT3 pathway: Crucial for mediating melatonin’s effect on microglia/macrophage polarization towards the M2 phenotype, thus reducing neuroinflammation [72]. The upstream JAK2 kinase is also involved [71].
  ○ Nrf2/HO-1 pathway: Melatonin activates the transcription factor Nrf2, which induces the expression of antioxidant enzymes like heme oxygenase-1 (HO-1), SOD, and GPx, bolstering cellular defenses against oxidative stress [74].
  ○ AMPK/mTOR pathway: Melatonin modulates this pathway, which regulates cellular energy homeostasis, autophagy, and protein synthesis. In neonatal hypoxia-ischemia, melatonin’s neuroprotection was associated with regulation of AMPK/mTOR/autophagy signaling [75]. Melatonin may also enhance autophagy via PI3K/Akt/mTOR in other injury models [69].
  ○ NF-κB pathway: As mentioned, melatonin inhibits NF-κB activation, a critical step in reducing the expression of pro-inflammatory genes, including iNOS, COX-2, and various cytokines, thereby limiting the inflammatory cascade after stroke [9].
  ○ MAPK pathways: Melatonin can modulate MAPK signaling. For instance, inhibition of p38 MAPK contributes to its suppression of iNOS in glial cells [43]. Modulation of ERK pathways has also been implicated in melatonin’s actions, sometimes promoting survival [61].

The convergence of melatonin and NO-related signals onto these pathways highlights the integrated nature of cellular responses to I/R injury and melatonin’s ability to orchestrate a multi-pronged protective strategy. The prominent role of mitochondria as both a source of damaging species (ROS and RNS) and a target of melatonin’s protective actions underscores the importance of mitochondrial integrity in determining cell fate after stroke. Melatonin’s capacity to accumulate in mitochondria [36] positions it ideally to counteract mitochondrial dysfunction driven by oxidative/nitrosative stress. Similarly, the modulation of microglia polarization represents a specific and significant mechanism by which melatonin targets the detrimental inflammatory component of stroke pathology, a process intimately linked with iNOS activity.

## 4. Conclusions

### 4.1. Main Findings

This review has comprehensively examined the distinct physiological roles of melatonin and nitric oxide (NO), as well as their intricate interplay, in the pathophysiology of ischemic stroke. A central focus has been the elucidation of the melatonin–NO system axis and its significance in understanding and potentially treating this devastating neurological condition. The evidence synthesized underscores melatonin’s multifaceted neuroprotective capacity, stemming from its potent antioxidant, anti-inflammatory, and anti-apoptotic properties. These attributes contribute to observed benefits such as reduced infarct volume and brain edema, improved neurological outcomes, and preservation of blood–brain barrier integrity following ischemic events. Indeed, melatonin’s pleiotropic actions, targeting multiple interconnected pathological pathways including the mitigation of neuroinflammation and excitotoxicity, position it as a highly promising agent in the context of cerebral ischemia-reperfusion injury.

Conversely, nitric oxide presents a more complex, dual role in ischemic stroke. Its effects are highly dependent on the generating synthase isoform, local concentration, and temporal context. eNOS-derived NO typically confers neuroprotection by maintaining vascular homeostasis and reducing inflammation, whereas NO produced by nNOS and iNOS often exacerbates neuronal damage through mechanisms like peroxynitrite formation and oxidative stress. This dichotomy highlights the necessity for nuanced therapeutic strategies rather than indiscriminate modulation of total NO levels.

Crucially, this review illuminates the pathophysiological significance of the melatonin–NO system axis, suggesting that melatonin’s neuroprotective actions are, at least in part, mediated through its modulation of the NO system. Melatonin has been shown to reduce overall NO production during ischemia and can influence the expression and activity of NOS isoforms, potentially shifting the NO balance towards neuroprotection by mitigating the detrimental effects of nNOS-derived NO. This interaction implies that melatonin may act as a critical re-balancer of NO homeostasis in the ischemic brain, an effect that appears sensitive to the timing of its administration.

The interplay within the melatonin–NO axis extends beyond acute neuroprotection, with implications for long-term neuronal integrity and functional recovery, including processes vital for synaptic plasticity, learning, and memory. Therefore, a deeper understanding of this axis not only clarifies fundamental mechanisms of ischemic brain injury, but also strengthens the rationale for developing therapeutic interventions that specifically target the interaction between melatonin and the NO system, offering a potentially more refined approach to ameliorate the consequences of ischemic stroke.

### 4.2. Recap of Key Mechanisms

The neuroprotective efficacy of melatonin in animal models of stroke, mediated significantly through its interaction with the NO system, relies on a multifaceted mechanism of action:

- Differential NOS isoform regulation: Suppression of detrimental iNOS, preservation/enhancement of protective eNOS, and variable modulation of nNOS shifts the NO balance towards neuroprotection.
- Direct RNS scavenging: Potent neutralization of NO and, critically, ONOO^−^, prevents downstream molecular damage.
- Mitochondrial protection: Melatonin safeguards mitochondria from NO/ONOO^−^-mediated damage, preserving energy production, reducing ROS leakage, and inhibiting apoptotic pathways.
- Signaling pathway modulation: Activation of pro-survival pathways (e.g., Akt/PI3K and Nrf2/HO-1) and inhibition of pro-inflammatory/pro-death pathways (e.g., NF-κB and p38 MAPK) integrate melatonin and NO signals.
- Anti-inflammation via microglia polarization: Melatonin promotes a shift from pro-inflammatory M1 to anti-inflammatory M2 microglia/macrophages, partly via STAT3 signaling, dampening the damaging inflammatory response.

## 5. Future Perspectives

### 5.1. Therapeutic Potential

The extensive and consistent preclinical data demonstrating melatonin’s ability to reduce infarct volume, decrease edema, improve functional outcomes, and target multiple key pathological pathways (oxidative stress, inflammation, apoptosis, and mitochondrial dysfunction) strongly support its potential as a therapeutic agent for ischemic stroke [6]. Its favorable safety profile observed in human studies for other indications, coupled with its ability to readily cross the BBB, further enhances its clinical appeal [6]. Targeting the melatonin–NO axis specifically, either through melatonin administration, potentially in combination with other therapies (like thrombolytics [76] or oxygen [51]), or via the development of novel melatonin analogs with optimized modulatory effects on NOS isoforms and related pathways, represents a promising avenue for future stroke treatment strategies.

### 5.2. Knowledge Gaps and Future Directions

Despite the compelling evidence, several areas require further investigation to fully understand the melatonin–NO interaction in the CNS and translate preclinical findings into effective stroke therapies:

- Mechanistic precision: Further studies are needed to dissect the precise molecular mechanisms by which melatonin differentially regulates nNOS and eNOS activity and expression in specific neuronal, glial, and endothelial cell types within the CNS, particularly during different phases of stroke evolution (acute, subacute, and chronic).
- Receptor vs. non-receptor roles: The relative contributions of melatonin’s receptor-mediated actions (via MT1/MT2) versus its direct, receptor-independent scavenging and mitochondrial effects in mediating specific aspects of NO modulation and neuroprotection in stroke need further clarification. Studies utilizing specific receptor antagonists (like luzindole [25]) or agonists, and potentially receptor knockout models (while considering endogenous melatonin levels), are warranted.
- Signaling network integration: A deeper understanding of how signaling pathways like Akt, STAT3, NF-κB, Nrf2, and AMPK/mTOR integrate signals from both melatonin and the NO system to orchestrate the overall cellular response to ischemic injury is required.
- Translational considerations: Rigorous clinical trials are essential to confirm the efficacy, optimal dosing, administration route, and therapeutic window for melatonin in human stroke patients [23]. Potential species differences in melatonin metabolism, receptor pharmacology [25], and NOS regulation [77] must be carefully considered during translation. The issue of endogenous melatonin deficiency in common laboratory mouse strains also highlights the need for appropriate model selection and interpretation [78]. The development of controlled-release or brain-targeted melatonin formulations could enhance its therapeutic efficacy and minimize side effects.
- Role of metabolites: The contribution of melatonin metabolites, such as AFMK and AMK, which possess significant antioxidant properties, to the overall neuroprotective effects observed in the CNS and stroke models warrants further investigation [19].
- Endogenous vs. exogenous melatonin: Distinguishing the protective roles of basal endogenous melatonin levels versus the effects of pharmacological administration is important, particularly given the observed decline in melatonin with age and in certain disease states, including potentially stroke [73].

Addressing these knowledge gaps will be crucial for optimizing the potential therapeutic application of melatonin and harnessing the beneficial aspects of the melatonin–NO interaction for the treatment of ischemic stroke and potentially other neurological disorders involving nitrosative stress and inflammation.
